# Optimization of substation grounding grid design for horizontal and vertical multilayer and uniform soil condition using Simulated Annealing method

**DOI:** 10.1371/journal.pone.0256298

**Published:** 2021-09-07

**Authors:** Navinesshani Permal, Miszaina Osman, Azrul Mohd Ariffin, Navaamsini Boopalan, Mohd Zainal Abidin Ab Kadir

**Affiliations:** 1 Institute of Power Engineering, Universiti Tenaga Nasional, Kajang, Selangor, Malaysia; 2 Department of Electrical and Electronics Engineering, University Tenaga Nasional, Kajang, Malaysia; 3 Centre for Electromagnetic and Lightning Protection (CELP), Advanced Lightning, Power, and Energy Research Centre (ALPER), Universiti Putra Malaysia, Seri Kembangan, Selangor, Malaysia; Torrens University Australia, AUSTRALIA

## Abstract

Grounding systems are critical in safeguarding people and equipment from power system failures. A grounding system’s principal goal is to offer the lowest impedance path for undesired fault current. Optimization of the grounding grid designs is important in satisfying the minimum cost of the grounding system and safeguarding those people who work in the surrounding area of the grounded installations. Currently, there is no systematic guidance or standard for grounding grid designs that include two-layer soil and its effects on grounding grid systems, particularly vertically layered soil. Furthermore, while numerous studies have been conducted on optimization, relatively limited study has been done on the problem of optimizing the grounding grid in two-layer soil, particularly in vertical soil structures. This paper presents the results of optimization for substation grounding systems using the Simulated Annealing (SA) algorithm in different soil conditions which conforms to the safety requirements of the grounding system. Practical features of grounding grids in various soil conditions discussed in this paper (uniform soil, two-layer horizontal soil, and two-layer vertical soil) are considered during problem formulation and solution algorithm. The proposed algorithm’s results show that the number of grid conductors in the X and Y directions (N_x_ and N_y_), as well as vertical rods (N_r_), can be optimized from initial numbers of 35% for uniform soil, 57% for horizontal two-layer soil for ρ_1>_ ρ_2,_ and 33% for horizontal two-layer soil for ρ_1<_ ρ_2_, and 29% for vertical two-layer soil structure. In other words, the proposed technique would be able to utilize square and rectangle-shaped grounding grids with a number of grid conductors and vertical rods to be implemented in uniform, two-layer horizontal and vertical soil structure, depending on the resistivity of the soil layer.

## Introduction

A well-designed grounding system is an essential factor in ensuring a safe operation of electrical systems which plays a major role in ensuring electromagnetic affinity, human protection and wellbeing, and the reliability of devices. The goal of an ideal grounding system is to reduce construction costs and time involved while satisfying the safety threshold parameters. The safety threshold parameters which consist of the grid impedance, step, and touch voltages are used to measure the behavior and safety level of a grounding system. These safety threshold parameters are computed using the equations from IEEE 80 [1]. For a grounding system to be declared safe, the magnitudes of the safety parameters must be below the threshold values. The safety threshold parameters are affected by the grounding grid geometry, grounding design parameters, and the electrical properties of the soil on which the grounding system is installed. Computerized grounding analysis in uniform and two-layer soil categories has become extensive, owing to the enhanced precision, speed, and adaptability provided by computer use. To address the optimization challenge, the traditional trial-error approaches become extremely time-consuming. Therefore, some non-traditional probabilistic search algorithms like the Genetic Algorithm (GA) [2–4], Particle Swarm Optimization (PSO) algorithm [4, 5], and Harmony Search (HS) algorithm [6], have been used to optimize grounding systems in recent years. Table 1 shows the various algorithms that are built to optimize grounding impedance, increase grounding effectiveness, and reduce engineering costs. Most of the optimization was conducted in uniform and horizontal two-layer soil structure but none was analyzed in vertical soil structure due to the complex nature of the computation involved.

**Table 1:** Optimization methods used in the grounding system.

Optimization method	Soil structure	References
IEEE & Finite Element Analysis (FEM) method using ETAP 12.60 software	Not mentioned	[15]
Gravitational Search Algorithm (GSA), Particle Swarm Algorithm (PSO)	Uniform	[5]
IEEE method using ETAP software	Horizontal two-layer	[16]
Optimal Compression Ratio (OCR) & fitting function	Horizontal two-layer	[17]
Quasi-static composite image method & Green function, dual-port model	Uniform	[10]
Hybrid method using chemical electrolyte ground rods, auxiliary wire mats, and ground enhancing material	Horizontal two-layer	[24]
Simulated Annealing (SA)	Uniform	[23]
Particle swarm optimization (PSO), space variable technique & harmony search algorithm (HS) using MATLAB	Uniform	[6]
Genetic algorithm (GA), multi-objective particle swarm optimization (MOPSO) algorithm, Bees, IEEE, and Schwarz’s equation	Uniform	[4]
Optimum Compression Ratio (OCR), Particle Swarm Optimization (PSO)	Horizontal two-layer	[22]
Steepest Descent Method using CDEGS	Horizontal two-layer	[25]
Variable spacing techniques created OPTIMA function in MATLAB	Horizontal two-layer	[18]
Economical Substation Grounding System Designer using MATLAB	Uniform	[26]
ATP- EMTP & genetic algorithm	Uniform	[19]
Genetic algorithm optimization	Uniform	[20]
Simulated Annealing, Optimized Finite Element Method	Uniform	[9]

Many researchers have attempted to address the grounding design optimization problem using various optimization techniques [4, 7–14]. Analytical and heuristic approaches are the most common strategies employed. Table 2 shows the pros and cons of various optimization techniques. Analytical techniques in [15–18] are presented to address optimal grounding system configuration issues. Analytical techniques have the disadvantage of being unable to solve multi-objective optimization issues. To address the grounding system challenge, several researchers employed the GA method [2, 4, 19, 20]. Wesley et al. [2] proposed a method by using GA to acquire soil characteristics that may be expressed in a multilayer structure. The method is based on an apparent resistivity curve generated from soil resistivity measurements. However, GA is poor in tackling the problem of a restricted local search space, is easily overestimated, and relies on the initial population for its solution. Besides, the grounding system optimization problem in [6] is solved using the HS algorithm. However, it has a low convergence rate and a low precision of optimization. The HS algorithm could not directly deal with constraints, thus a variety of constraint-handling approaches should be employed to help with the optimization process [13, 21]. Furthermore, the PSO method [5, 6, 22], is utilized to optimize the design parameters of the grounding system, however the method has difficulty with discrete optimizations. The PSO algorithm has a weakness since it converges prematurely after being trapped in local optima, which it mistook for global optima. When the PSO algorithm is used to a complicated multi-dimensional situation, it becomes difficult to escape the local optima and achieve the global optima due to specific restrictions. The SA method is used to solve the grounding design parameters issue in [9, 23]. However, it has a slow convergence speed and parallel computing is challenging.

**Table 2:** Advantages and drawbacks of various algorithms in grounding design optimization.

Algorithm	Advantages	Drawbacks	Refs.
Analytical method	The approach is easy and requires minimal work.	Only deals with optimization problems with a single-objective	[15–18]
Genetic Algorithm (GA)	The convergence speed is fast and the versatility is strong	Complex, poor at handling the problem of restricted local search space, easy to overestimate, and its solution is dependent on the initial population	[2, 4, 19, 20]
Particle Swarm Optimization (PSO)	Has a good memory, fast training speed, simple method	It is easy to fall into local optimal, inadequate discrete optimization issue management	[5, 6, 22],
Harmony Search (HS)	Reasonable execution time, simple	The convergence that occurs too soon	[13, 21]
Simulated Annealing (SA)	Strong global search capability	Convergence speed is slow, parallel computing is challenging	[9, 23]

From all of the optimization methods that have been compared, the SA algorithm is proposed in this paper due to the discrete nature of grounding design parameters and the complexity of the problem which will increase with complex soil properties in which the grounding grid is installed. As a result, the optimization process requires a strong global search capability, with the SA algorithm being able to avoid early convergence to local optima and diverge the solution when compared to other optimization methods. The proposed algorithm is validated against results simulated using the Current Distribution, Electromagnetic Fields, Grounding and Soil Structure Analysis (CDEGS) software and optimization techniques in Refs. [27] and [26].

## Input & methodology for grounding design parameters optimization

### Soil apparent resistivity

As previously stated, the ability of this optimization method for grounding design parameters to find an optimal solution in a variety of soil conditions is its main significance. The soil’s apparent resistivity is one of the most critical soil characteristics that influence the performance of a grounding system under various soil conditions. The soil constants and empirical formulas of the uniform soil, horizontal two-layer soil, and vertical two-layer soil model used to calculate the apparent resistivity are based on data available in [28, 29], as seen in Tables 3 and 4. The calculated apparent resistivity will be utilized as the input for the optimization process of the grounding system in different soil conditions.

**Table 3:** Empirical formulas for apparent resistivity in different soil conditions [28].

Horizontal two-layer soil		
Soil condition	Soil Constant	Equations
*ρ*_2_<*ρ*_1_(-K)	1<M1≤3	$\rho_{a}=\frac{\rho_{1}}{\left[1+\left[(\frac{\rho_{1}}{\rho_{2}})-1\right][1-e^{\frac{M1}{K(0.9h_{g}+0.9h_{top})}}]\right]}$
*ρ*_2_<*ρ*_1_(-K)	3<M1≤5	$\rho_{a}=\frac{\rho_{1}}{\left[1+\left[(\frac{\rho_{1}}{\rho_{2}})-1\right][1-e^{\frac{M1}{K(1.9h_{g}+1.9h_{top})}}]\right]}$
*ρ*_2_<*ρ*_1_(-K)	M1>5	$\rho_{a}=\frac{\rho_{1}}{\left[1+\left[(\frac{\rho_{1}}{\rho_{2}})-1\right][1-e^{\frac{M1}{K(4.5h_{g}+4.5h_{top})}}]\right]}$
*ρ*_1_<*ρ*_2_(+K)	1<M2≤3	$\rho_{a}=\rho_{1}x[1+\left[(\frac{\rho_{2}}{\rho_{1}})-1\right]\left[1-e^{\frac{-1}{K*M2(1.5h_{g}+1.5h_{top})}}\right]]$
*ρ*_1_<*ρ*_2_(+K)	3<M2≤5	$\rho_{a}=\rho_{1}x[1+\left[(\frac{\rho_{2}}{\rho_{1}})-1\right]\left[1-e^{\frac{-1}{K*M2(2.1h_{g}+2.1h_{top})}}\right]]$
*ρ*_1_<*ρ*_2_(+K)	M2>5	$\rho_{a}=\rho_{1}x[1+\left[(\frac{\rho_{2}}{\rho_{1}})-1\right]\left[1-e^{\frac{-1}{K*M2(4.5h_{g}+4.5h_{top})}}\right]]$
**Vertical two-layer soil**		
$\rho_{a}=a\rho_{1}(\frac{1}{a}+\frac{k}{\sqrt{4d^{2}+4da\;\cos\;\beta +a^{2}}}+\frac{k}{\sqrt{4(d+3a\;\cos\;\beta )(d+2a\;\cos\;\beta )+a^{2}}}-\frac{k}{\sqrt{4d^{2}+8da\;\cos\;\beta +{4a}^{2}}}-\frac{k}{\sqrt{4(d+3a\;\cos\;\beta )(d+a\;\cos\;\beta )+{4a}^{2}}}$		
**Uniform soil**		
*ρ*_*a*_ = *ρ*_1_		

**Table 4:** Soil constant for horizontal two-layer soil model [28].

Soil condition	For *ρ*_2_<*ρ*_1_	For *ρ*_1_<*ρ*_2_
Soil constant	M1 = $\frac{\rho_{1}}{\rho_{2}}$	M2 = $\frac{\rho_{2}}{\rho_{1}}$

### Simulated Annealing algorithm

The Simulated Annealing algorithm is an intelligent optimization tool, explicitly tailored towards the optimization problem and has a series of benefits relative to the conventional optimization approach. The significance of the results is a set of good solutions instead of a single solution. It offers an alternate benefit, so it is particularly ideal for coping with difficult nonlinear engineering optimization issues. Simulated Annealing is a probabilistic search algorithm for an iterative adaptive heuristic that could be used to solve various nonlinear issues and compensate for non-differentiable and sometimes intermittent features. The probability of Simulated Annealing algorithms on achieving optimal solution could be higher. The optimal global solution can be achieved, with increased precision, global convergence, conditional parallel processing, and broad adaptability, which can accommodate multiple types of variables in optimization without any secondary knowledge on the objective function and thus no specifications for the control function.

To begin with a simple grounding model, the design parameters in the analysis of grounding performance are usually chosen in the general order or based on the physical grounding system available at the site. As a general practice, the number of grid conductors in the X and Y directions (N_x_ and N_y_) as well as vertical rods (N_r_), are increased to obtain a lower grounding impedance and below threshold values for step and touch voltages. A design engineer might use as many grid conductors and vertical rods of different lengths as possible to protect a grounding system. However, a good grounding system should not be not only efficient but also cost-effective. Therefore, an optimization algorithm to reduce the engineering costs of a grounding system, easy modelling of grounding system in any engineering software, and lesser computation time is developed using the Simulated Annealing (SA) method. The limitations of grounding grid design parameters and safety thresholds are referred to from relevant equations and standard data from IEEE 80 [1] for developing the optimization algorithm. The design parameters of the grounding grid consist of the X-direction number of horizontal conductors, (N_x_) and Y-direction number of horizontal conductors (N_y_), and the number of vertical rods, (N_r_).

## Problem formulation

### Objective function

The purpose of grounding design optimization is to reduce the objective function, which is material and installation costs while maintaining safety. The cost of the grounding grid system can be broken down into four major components, with the cost function computed using (1) [6].

1. Cost of grounding grid conductor (C_1_), it is associated with the size and length of the grid conductor.
2. Cost of burying the conductor (C_2_), it is associated with the depth of grid buried, length of grid conductor
3. Cost of welding (C_3_), associated with the number of conductors on each side.
4. Cost of rods (C_4_), it is associated with the number of vertical rods.

$$Cost.Func.(f_{c})=C_{1}.A.(N_{x}.L_{y}+N_{y}.L_{x})+C_{2}.h.(N_{x}.L_{y}+N_{y}.L_{x})+C_{3}.(N_{x}.N_{y})+{C_{4}.(N}_{r})$$ (1)

Where,

A is the total area enclosed by the ground grid, m^2^; N_x_ is the number of grid conductors in x-direction; N_y_ is the number of grid conductors in y-direction; L_x_ is the length of grid conductor in x-direction; L_y_ is the length of grid conductor in y-direction; h is the depth of grid conductor; N_r_ is the number of rods.

### Safety constraints

#### Computation of safety threshold parameters

A stable grounding system should be capable of sustaining the actual mesh and step voltages under the equivalent tolerable level within a substation. Equivalent grounding system impedance should be sufficiently low to ensure the more fault current disperses across the grounding grid into the earth to allow the grounding systems to be more secure. The optimization algorithm presented in this paper can be implemented for both 50 kg and 70 kg of body weight, although the results presented in this paper only demonstrated the step and touch voltages calculated for 50 kg only as an example for a worst-case scenario. The main focus should be given to the detail wherein any circumstances, the actual mesh and step voltages must not surpass the threshold values given in (2) and (3) [1] which define the maximum value of voltages that human beings can withstand in the case of an accidental step voltage and an accidental touch voltage scenario when designing the grounding system.


$${E_{step50thres}=(1000+6.\rho }_{s.}C_{s.}).\frac{0.116}{\sqrt{t_{s}}}$$ (2)

$${E_{touch50thres}=(1000+1.5.\rho }_{s.}C_{s.}).\frac{0.116}{\sqrt{t_{s}}}$$ (3)

Where E_step50thres_ = tolerable step voltages for a person weighing 50 kg; E_touch50thres_ = tolerable touch voltages for a person weighing 50 kg; E_s_ = calculated step voltages; E_m_ = calculated mesh voltages

**Fig 1:**
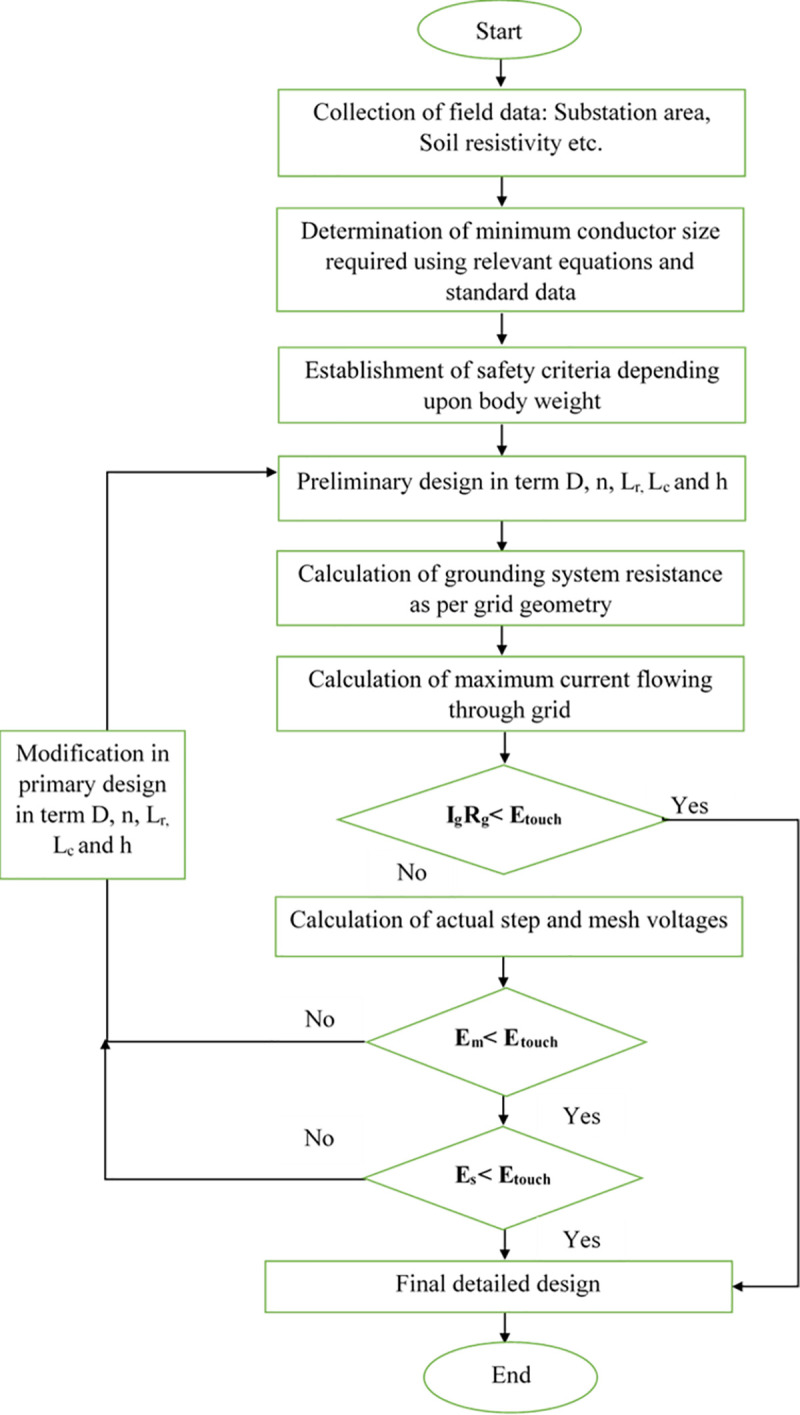
Flowchart of grounding system design methodology according to IEEE 80 [1].

For the grounding system to be protected, the measured voltages present on substation earth as in (4) and (5) and grounding resistance value calculated using (6) must be lower than the threshold values provided by (2) and (3) for voltages and 5Ω for grounding resistance. Fig 1 displays a flowchart explaining the IEEE Grounding System Design approach. Detailed instructions and related equations for the design of the grounding system are available from [1].


$$E_{s}=\frac{\rho_{a}+K_{i}+K_{s}+I_{g}}{Ls}$$ (4)

$$E_{m}=\frac{\rho_{a}+K_{i}+K_{m}+I_{g}}{Lm}$$ (5)

$$R_{g}=\rho_{a}\left[\frac{1}{L_{t}}+\frac{1}{\sqrt{20A}}(1+\frac{1}{1+h(\sqrt{20/A)}}\right]$$ (6)

Where

*ρ*_*s*_ = soil surface resistivity (Ω.m)

*t*_*s*_ = duration of a shock for determining allowable body current (s)

C_s_ = surface layer de-rating factor

*ρ* = soil resistivity (Ω.m)

L_t_ = total length of conductors in the grounding system (m)

I_g_ = maximum grid current (A)

K_i_ = correction factor of grid geometry

K_s_ = step voltage spacing factor

L_s_ = total length of conductor for a step voltage (m)

K_m_ = touch voltage spacing factor

L_m_ = total length of conductor for touch voltage (m)

h = depth of grounding grid buried (m)

A = area of the grounding system (m^2^)

D = spacing between conductors (m)

n = geometric factors of grounding

The limitations of R_g,_ E_step_ and E_m_ are determined in (7)–(9) by the protection criterion of equipment and systems concerning the remote earth which usually considers the insulation level and the performance of protection devices and systems. In most substations, R_g_ is in the range of 5 Ω. Based on the above descriptions, the safety constraints can be expressed as the following formulae:

$$E_{m}\leq E_{touch50thres}$$ (7)

$${E_{s}\leq E}_{step50thres}$$ (8)

$$R_{g}\leq 5Ω$$ (9)

#### Solution algorithm

**Fig 2:**
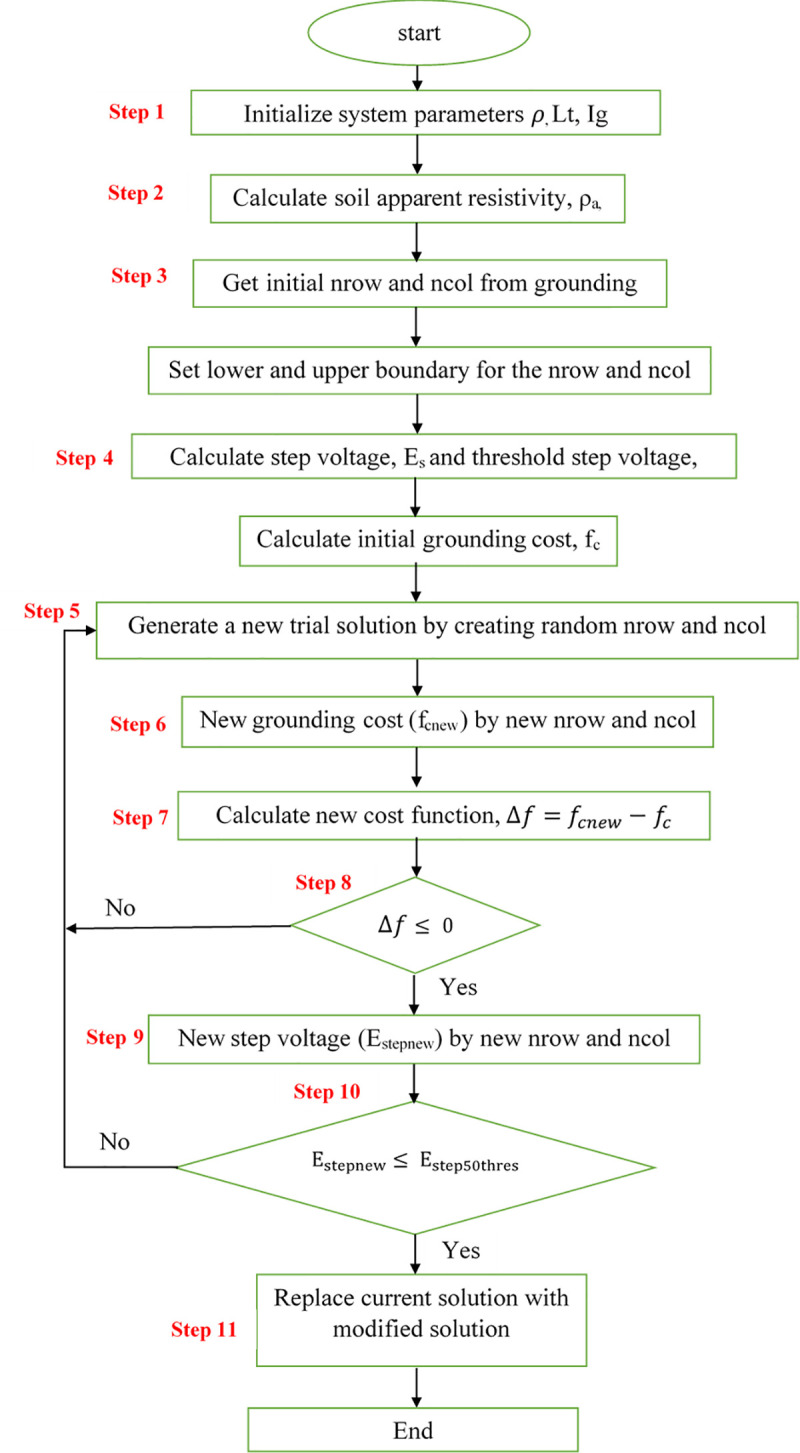
Flowchart of grounding system design optimization using Simulated Annealing algorithm.

The use of the Simulated Annealing algorithm to lessen the design cost while ensuring grounding grid safety is shown in Fig 2. The following Steps 1–11 are the details of the grounding optimization processes of the Simulated Annealing algorithm. These steps are similarly applied to calculate the touch voltage and grid impedance using relevant equations.

**Step 1.** Input system data

Set the number of soil layers, soil resistivity (*ρ*) for uniform soil, soil resistivity (*ρ*_1_) and (*ρ*_2_) for two-layer soil, the total length of the grid and rod conductors (L_t_), depth of grid (h), fault current (I_g_), and so on.

**Step 2.** Calculate the apparent resistivity of the soil (*ρ*_*a*_) using the equations in Tables 3 and 4 according to the soil structures where a grounding system will be placed.
**Step 3.** Set the initial configuration of a grounding grid.

X-direction number of horizontal conductors, N_x_ (nrow) and Y-direction number of horizontal conductors, N_y_ (ncol) of a grounding grid design. Set the lower [lb] and upper boundary [ub] for the nrow and ncol of the grounding grid.

**Step 4.** Calculate safety threshold parameters and initial cost function

Step voltage threshold value (*E*_*step*50*thres*_) is calculated using (3) while the step voltage of the current grounding system (*E*_*s*_) using (5). The initial grounding cost (f_c_) is calculated using (2).

**Set 5.** Generate a feasible solution

A new trial solution of ncol and nrow will be generated randomly, ncol and nrow are random numbers between [lb, ub].

**Step 6**. Calculate *f*_*cnew*_ using new solutions of ncol and nrow.
**Step 7.** Calculate cost function, Δf = *f*_*cnew*_−*f*_*c*_
**Step 8.** Check loop criterion

If Δf is ≤ 0, the current solution is approved and the optimal solution estimation is changed to the adjusted solution. Otherwise, the existing solution is unchanged. 0 is the threshold of function tolerance which if it is exceeded, the iterations of the solver will stop and move on to the next step.

**Step 9.** Check safety constraints

Step voltage of the current grounding system (*E*_*s*_) is calculated using the new solution of nrow and ncol and compared with (*E*_*step*50*thres*_). If the constraint is violated, go to Step 5. Otherwise, proceed to the next step.

**Step 10.** Check stop criterion

If *E*_*stepnew*_≤*E*_*step*50*thres*_ the current solution is approved and the optimal solution estimation is changed to the adjusted solution. Otherwise, the existing solution is unchanged. Repeat Steps 5–10 above, until loop iteration steps fulfill the criteria.

**Step 11.** Output the optimal configuration

## Results & discussions

### Validation of the Simulated Annealing algorithm

Various parameters have many impacts on the efficiency of the grounding system. The X-direction (N_x_), and Y- direction (N_y_) number of grid conductors, area of the grid (A), number of vertical rods attached to the main grid (N_r_), and spacing between conductor’s burial depth (h) were defined as the most prevalent and controllable parameters affecting the output of the grounding system [30]. A detailed explanation of the response of these parameters on the efficiency of the grounding system can be found in [31]. Up to two of these parameters can be the changing variables when deciding on the optimization issue of grounding system design. Optimization is implemented to make the design reliable and cost-effective in such a manner that the technological efficiency of the grounding system in terms of human protection and safety of equipment is never compromised and additional cost advantages are incurred by meeting all the essential electrical safety specification criteria.

To validate the results given by the SA algorithm, a simple design problem is solved using Matlab software and the MALZ module of CDEGS which is one of the grounding and electromagnetic analysis software packages available. The input data of the grounding system design parameters for uniform and two-layer soil structures are shown in Table 5 is adapted from [26]. Tables 6 and 7 provide further information on the soil characteristics of horizontal and vertical two-layer soil structures respectively based on assumptions, as Ref [26] only presented a uniform soil structure. As a consequence, the uniform soil structure findings in Table 8 are validated using both CDEGS and Ref [26] whereas the results in Tables 9–12 are validated between SA and CDEGS.

**Table 5:** Grounding system design input for uniform and horizontal two-layer soil structure [26].

Design Parameters	Values
Grid area (A)	63 m x 33 m
Depth of grid burial (h)	0.6 m
Number of conductors in the X direction (N_x_)	11
Number of conductors in the Y direction (N_y)_	20
Spacing between conductors (D)	3.3 m
No. of. ground rods (N_r_)	17
Length of ground rod (L_r_)	3 m
Uniform soil resistivity (ρ)	40 Ω.m
Fault current (I_f_)	25 kA
Surface layer resistivity (ρ_s_)	3000 Ω.m
The thickness of the surface layer (h_s_)	0.1 m
Shock duration (t_s_)	0.5 s

**Table 6:** Soil properties of horizontal two-layer soil structure.

Upper layer soil resistivity (ρ_1_)	40 Ω.m
Lower layer soil resistivity (ρ_2_)	20 Ω.m
Upper layer soil height (h_g_)	5 m

**Table 7:** Soil properties of vertical two-layer soil structure.

Design Parameters	Values
Distance between four electrodes (*a*)	5 m
Angle between line where four electrodes are located and perpendicular line to the boundary between layers 1 and 2 (*β*)	60^0^
Normal distance between the first electrode and boundary between layers 1 and 2 (*d*)	10 m
Right layer soil resistivity (ρ_1_)	40 Ω.m
Left layer soil resistivity (ρ_2_)	20 Ω.m

**Table 8:** Results for grounding system in uniform soil.

Particular	ρ = 40 Ω.m			% difference SA-CDEGS	% difference SA- Ref [26]
	SA	CDEGS	Ref [26]		
Resistance (R_g_)	0.39 Ω	0.36 Ω	0.4099 Ω	7.69	4.85
E_step_ threshold	2212.70 V	2146.88 V	2212.7 V	2.97	0
E_touch_ threshold	676.22 V	652.65 V	676.22 V	3.49	0
E_step_ calculated	787.60 V	832.54 V	710.32 V	5.39	10.89
E_touch_ calculated	690.7 V	771.45 V	452.39 V	10.46	52.67

**Table 9:** Results for grounding system in horizontal two-layer soil (ρ_1>_ ρ_2_).

Particular	ρ_1_ = 40 Ω.m; ρ_2_ = 20 Ω.m		% difference SA -CDEGS
	SA	CDEGS	
Resistance (R_g_)	0.24 Ω	0.23 Ω	4.1
E_step_ threshold	2207.70 V	2146.88 V	2.75
E_touch_ threshold	674.96 V	652.60 V	3.31
E_step_ calculated	683.11 V	673.26 V	1.46
E_touch_ calculated	1740.80 V	771.45 V	5.44

**Table 10:** Results for grounding system in horizontal two-layer soil (ρ_1<_ρ_2_).

Particular	ρ_1_ = 40 Ω.m; ρ_2_ = 80 Ω.m		% difference SA -CDEGS
	SA	CDEGS	
Resistance (R_g_)	0.55 Ω	0.58 Ω	5.45
E_step_ threshold	2207.70 V	2146.88 V	3.06
E_touch_ threshold	674.96 V	652.60 V	3.56
E_step_ calculated	1050.60 V	1039.23 V	1.09
E_touch_ calculated	3040.40 V	2871.96 V	5.86

**Table 11:** Results for grounding system in vertical two-layer soil (ρ_1>_ ρ_2_).

Particular	ρ_1_ = 40 Ω.m; ρ_2_ = 20 Ω.m		% difference SA -CDEGS
	SA	CDEGS	
Resistance (R_g_)	0.23 Ω	0.25 Ω	8.01
E_step_ threshold	2208.1 V	2138.34 V	3.15
E_touch_ threshold	675.10 V	650.46 V	3.65
E_step_ calculated	652.25 V	627.35 V	3.82
E_touch_ calculated	903.28 V	856.76 V	5.15

**Table 12:** Results for grounding system in vertical two-layer soil (ρ_1<_ ρ_2_).

Particular	ρ_1_ = 40 Ω.m; ρ_2_ = 80 Ω.m		% difference SA -CDEGS
	SA	CDEGS	
Resistance (R_g_)	0.45 Ω	0.49 Ω	8.16
E_step_ threshold	2215.8 V	2163.83 V	2.35
E_touch_ threshold	677.05 V	656.84 V	2.99
E_step_ calculated	1304.5 V	1241.49 V	4.83
E_touch_ calculated	1206.6 V	1128.75 V	6.45

From the tables, there is a close agreement with the results of both the calculated and threshold values of step and touch voltages obtained by the SA algorithm and CDEGS. It can be seen that the percentage error between the SA algorithm and CDEGS is in the acceptable range of 2.97% to 10.7% for uniform soil, 1.09% to 5.86% for horizontal two-layer soil, and 2.35 to 8.16% for vertical two-layer soil structure. As for the comparison between the SA algorithm and the results presented in Ref [26] for uniform soil, the percentage error is 0% for threshold values of step and touch voltages. The percentage error for the calculated step voltage and grid resistance is 4.87% and 10.89% respectively. A major difference can be seen for calculated touch voltage at 52.67% for comparison between Ref [26]. This could be due to the difference in the equation used in Ref [26] as the percentage error for touch voltage between CDEGS and SA is only 10%. Thus, this validates the Simulated Annealing algorithm’s performance developed using Matlab software as most of the percentage error falls within an acceptable range.

### Optimization of number of horizontal conductors in X and Y directions and number of vertical rods

The input parameters needed for the optimization of grounding system design in uniform soil are shown in Table 5. The soil resistivity for uniform soil is 40 Ω.m. Table 13 shows the corresponding results for optimizing the number of horizontal conductors (N_x_ and N_y_) and vertical rods (N_r_) in uniform soil structure. The cost of grounding is not included in this study since it is well known that using a less or optimal number of grid conductors and vertical rods can minimize grounding system implementation costs while ensuring system protection.

**Table 13:** Results for grounding system in uniform soil.

Particular	Results (SA)		Results (Ref [26])		
	Before optimization	After optimization	Before optimization	After optimization	% difference SA—Ref [26] After optimization
Resistance (R_g_)	0.39 Ω	0.35 Ω	0.4099 Ω	0.417 Ω	16.06
Estep calculated	787.6 V	548.91 V	710.32 V	609.03 V	9.87
Etouch calculated	690.7 V	668.17 V	452.39 V	573.32 V	16.54
N_x_	11	12	11	14	14.29
N_y_	20	13	20	8	62.5
N_r_	17	15	17	49	69.38

The grounding design optimization in horizontal two-layer soil structure is conducted using the input data of the grounding design parameters shown in Table 14 adapted from [27]. For two-layer soil model, ρ_1_ = 400 Ω.m; ρ_2_ = 200 Ω.m for ρ_1>_ ρ_2_, and ρ_1_ = 400 Ω.m; ρ_2_ = 800 Ω.m for ρ_1<_ρ_2_. The corresponding results for optimizing the number of horizontal conductors (N_x_ and N_y_) and vertical rods (N_r_) in horizontal two-layer soil structure are presented in Tables 15 and 16.

**Table 14:** Grounding system design input for horizontal two-layer soil structure [27].

Design Parameters	Values
Grid area (A)	70 m x 70 m
Depth of grid burial (h)	0.5 m
Number of conductors in the X direction (N_x_)	21
Number of conductors in the Y direction (N_y)_	21
Spacing between conductors (D)	3.5 m
Fault current (I_f_)	1800 A
Surface layer resistivity (ρ_s_)	2500 Ω.m
The thickness of the surface layer (h_s_)	0.1 m
Shock duration (t_s_)	0.5 s
Upper layer soil resistivity (ρ_1_)	400 Ω.m
Lower layer soil resistivity (ρ_2_)	200 Ω.m or 800 Ω.m
Upper layer soil height (h_g_)	10 m

**Table 15:** Results for grounding system in horizontal two-layer soil (ρ_1>_ ρ_2_).

Particular	Results (SA)		Results ([27])		
	Before optimization	After optimization	Before optimization	After optimization	% difference SA–Ref [27] After optimization
Resistance (R_g_)	1.70 Ω	1.60 Ω	1.67 Ω	1.81 Ω	11.60
Estep calculated	327.35 V	253.05 V	305.9 V	249.2 V	1.54
Etouch calculated	560.06 V	507.54 V	484.8 V	742.0 V	31.6
Total conductor length	2940 m	1260 m	2940 m	1100 m	14.55

**Table 16:** Results for grounding system in horizontal two-layer soil (ρ_1<_ρ_2_).

Particular	Results (SA)		Results ([27])		
	Before optimization	After optimization	Before optimization	After optimization	% difference SA–Ref [27] After optimization
Resistance (R_g_)	3.89 Ω	3.52 Ω	3.88 Ω	3.91 Ω	9.97
Estep calculated	453.21 V	247.96 V	441.1 V	229.7 V	7.95
Etouch calculated	704.44 V	526.42 V	693.2 V	633.1 V	16.85
Total conductor length	2940 m	1960 m	2940 m	2280 m	20.34

There is no research article on the optimization of the grounding system in the vertical soil layer available. Therefore, the results in Table 17 are given by comparing the results from SA and a CDEGS-simulated optimal grounding system.

**Table 17:** Results for grounding system in vertical two-layer soil (ρ_1>_ ρ_2_).

Particular	Results (SA)		Results (CDEGS)		
	Before optimization	After optimization	Before optimization	After optimization	% difference SA–CDEGS After optimization
Resistance (R_g_)	1.75 Ω	1.70 Ω	1.84 Ω	1.88 Ω	10.58
Estep calculated	477.26 V	458.91 V	381.97 V	435.88 V	5.01
Etouch calculated	491.47 V	539.81 V	659.10 V	665.41 V	18.86
Total conductor length	2940 m	2100 m	2940 m	2100 m	0

The optimum number of grid conductors and vertical rods determined from the results would help in lowering the cost of grounding system implementation while maintaining the system’s protection. Without jeopardizing the safety of the grounding system, the total amount of horizontal and vertical conductors has been reduced from initial numbers ranging from 9% to 35% for uniform soil, 57% for horizontal two-layer soil for ρ_1>_ ρ_2,_ and 33% for horizontal two-layer soil for ρ_1<_ ρ_2_, and 29% for vertical two-layer soil structure. These percentages refer to the number of horizontal conductors in the X and Y directions, as well as the number of vertical rods in the grounding system, being reduced from the initial number to the optimal number of conductors and rods. Besides, compared to the optimization results from Ref [26] for uniform soil, Ref [27] for horizontal two-layer soil, the optimization algorithm proposed in this paper gives better results in optimizing the number of horizontal conductors and vertical rods.

This algorithm can optimize the design of the grounding system according to the updated system, even if the characteristics of the power system network are changed, such as the fault current or shock duration. As previously said, this algorithm employs the SA method to determine the best grounding design. Therefore, the algorithm would utilize the initial grounding design parameters to search for the best solution locally.

## Conclusions

The overall cost of a grounding system was reduced by lowering the number of conductors in the X and Y directions, as well as the number of vertical rods connected to the grid, using the Simulated Annealing (SA) approach, without affecting the ground grid’s protection. The proposed algorithm would be able to utilize square and rectangle-shaped grounding grids with a number of grid conductors and vertical rods to be implemented in uniform, two-layer horizontal and vertical soil structure which is yet to be done in any other research. The SA algorithm will diverge the parameters and delay premature convergence to the local optimum using its excellent local search capacity. The algorithm can successfully improve the optimization technique’s convergence to prevent misleading the local optimum. Comparison has been made between the SA algorithm in Matlab and grounding analysis software (CDEGS) in different soil conditions, where the minimal differences between the two methods show the validity of the algorithm developed.
